# A Cure for Headache

**Published:** 1868-12

**Authors:** George Kennion


### A Cure for Headache.

BY GEORGE KENNION, M. D., F. R. C. P

I am desirous of bringing before the notice of the profession a very simple, and at the same time a very remarkable cure for many kinds of headache. I have not the least claim to the discovery of this remedy, nor, indeed, am I at all aware who was its originator; but I believe that it is unknown to the profession generally; and having used it for nearly twelve months in a very large number of cases, and very rarely without affording immediate relief, I am desirous of making it more generally known. I heard of it first from a gentleman whom I was attending last year, and who told me that he thought it was used by a French physician. If this should come under his notice, I hope that it may be the means of inducing him to drop his incognito, so that he may receive the thanks of many to whom he has hitherto been an unknown benefactor.

The remedy, as I have already observed, is simple; it is the bisulphide of carbon in solution. Its mode of application is no less simple. A small quantity of the solution (about two drams) is poured upon cotton wool, with which a small, wide-mouthed, glass-stoppered bottle is half filled. This, of course, absorbs the fluid, and when the remedy has to be used, the mouth of the bottle is to be applied closely (so that none of the volutile vapor may escape) to the temple, or behind the ear, or as near as possible to the seat of pain, and so held for from three to five or six minutes. After it has been applied for a minute or two a sensation is felt as if several leeches were biting the part; and after the lapse of two, three, or four minutes more, the smarting and pain become rather severe, but subside almost immediately after the removal of the bottle. It is very seldom that any redness of the skin is produced. The effect of this application, as I have said, is generally immediate. It may be re-applied, if necessary, three or four times in the day.

The class of headaches in which this remedy is chiefly useful is that which may be grouped under the wide term of "nervous." Thus neuralgic headache, periodic headache, hysterical headache, and even many kinds of dyspeptic headache, are almost invariably relieved by it; and although the relief of a symptom is a very different affair, of course, from the removal of its cause, yet no one who has witnessed (and who of us has not seen?) the agony and distress occasioned by severe and repeated headache but must rejoice in having the power of affording relief in so prompt and simple a manner.

As regards the *modus operandi* of this remedy, it is difficult, perhaps, to form a certain opinion; but I am disposed to attribute it to the sedative effect of the vapor of the bisulphide, absorbed through the skin, and acting upon the superficial nerves of the part to which it is applied. The remarks of M. Delpech (*Annales d'Hygiene*, Jan'y, 1863,) point out very clearly the remarkable prostration of the whole nervous system produced in workmen who, in certain manufactures, are exposed to the vapor arising from a solution of the bisulphide of carbon; and we can readily understand that a somewhat similar effect, upon a small scale, may be produced by the application of this vapor to a limited portion of the surface.

I always procure the bisulphide of carbon from Mr. Morson, the eminent chemist in Southampton Row, Bloomsbury, who will also furnish the bottle with which the vapor should be applied, and a wooden case—a very necessary adjunct, on account of the offensive smell of the bisulphide.—*British Medical Journal*, June 13, 1868.

